# A Co‐production Evaluation Tool Informed by Co‐production Workshops for Use in Evidence Synthesis Contexts

**DOI:** 10.1002/cesm.70065

**Published:** 2026-01-07

**Authors:** Meena Khatwa, Vanessa Bennett, Rachael C. Edwards, Lisa Richardson, Phuong Tu Nguyen, Sajid Saleem, Sylvia Chaires, Alison O'Mara‐Eves, Dylan Kneale

**Affiliations:** ^1^ EPPI Centre, UCL Social Research Institute University College London London UK; ^2^ Co‐production Collective, UCL Culture University College London London UK

## Abstract

**Aim:**

We aimed to co‐produce a tool for evaluating co‐production within evidence syntheses.

**Background:**

Participatory approaches are recommended to enhance the salience and quality of evidence syntheses, and there is an increasing onus on co‐producing evidence synthesis. Co‐production is a way of working where research generators, beneficiaries and other interest holders work in equal partnership and for mutual benefit.

**Methods:**

To develop our approach, we:

- Examined selected existing tools and frameworks that could be useful in evaluating co‐production
- Developed an initial tool that was then modified through input from co‐production workshops
- Piloted the tool and evaluation approach in a project as part of research involving co‐producing a logic model to support evidence syntheses.

**Results:**

The existing tools guidance and resources we examined were deemed to be oriented towards supporting the conduct and reporting of co‐production, rather than evaluating what happens and how. This provided a basis for co‐producing a new tool. A new tool was developed that captures our perspectives on: positionality and expertise; motivations and expected benefits; clarity of role and expectations; project involvement and contributions; value and recognition; skills, knowledge, and personal growth; relationships and networking; comfort, support, and accessibility; and decision‐making and power sharing. We reflected that the tool and process for administering the tool worked well, and we liked the process of collective sensemaking.

**Conclusions:**

We believe that the tool (which we refer to as the STRAPS tool – Synthesising Through Reflection And Participatory Sense‐making) could provide a useful resource and starting point to other review teams who wish to evaluate co‐production in their reviews and encourage others to share their experiences with us.

**Implications:**

Co‐production enhances the quality of evidence syntheses. Use of the STRAPS tool can help reviewers unpack the process by which this happens using a standardised approach.

## 1 Introduction

Co‐production of evidence syntheses is of increasing interest as a strategy to democratise the production of research, disrupt and level power hierarchies between the producers of research and the intended beneficiaries, and create better research [1, 2, 3]. Co‐production entails working according to a set of values and principles [4, 5, 6] where research generators and beneficiaries work in equal partnership and for mutual benefit [7]. These values and principles can reflect both what co‐production is meant to achieve (e.g., equality and shifts in power differentials; embracing a diversity of perspectives, characteristics and experiences in the production of research) and values around how co‐production should be conducted (e.g., enhancing accessibility and inclusivity; ensuring that research is conducted in a reciprocal and mutually beneficial way) [4].

The movement towards co‐production builds on a history of public involvement in the production of systematic reviews [8, 9]. Co‐production shares parallels with other forms of public involvement, although generally places a greater emphasis on co‐producers' power and role in shaping both the process of conducting the research as well as the research outputs [4].

The London Alliance for the Co‐production of Evidence Synthesis (LACES) is an evidence synthesis group that places co‐production, and the values and principles of co‐production, at the centre of its work. Based at UCL, the LACES group involves systematic review methodologists (the EPPI Centre and Health Economics Analysis and Research methods Team), specialists in Co‐production methodologies (the Co‐production Collective), and a range of co‐producers working together to synthesise evidence across health topics. Co‐production was incorporated as a central pillar in the work of LACES based on our previous evidence syntheses and research projects where it became a transformative process that enhanced the quality of research [4, 10, 11]. While previous experiences have led us to identify the potential of co‐production in enhancing the quality, credibility, and salience of systematic reviews, we had not previously developed an approach to evaluating the processes and outcomes of co‐production in the context of evidence synthesis projects. Previous studies have explored ways of describing and understanding participatory approaches more generally within reviews [9] and research more broadly [12], although the transformatory nature of co‐production has prompted us to consider a tool more focussed on co‐production within evidence syntheses, that is itself co‐produced.

In this brief report, we present the co‐produced reflective tool and approach that we are using to evaluate the difference that co‐production makes to our evidence syntheses. We hope that this can serve as an aid to other systematic review teams. The tool involves administering a short survey of open‐ended questions, around which reflective workshops can be held. The tool was co‐produced as part of research involving co‐producing a logic model to support evidence syntheses.

## 2 Methods

1. **Examining existing tools and frameworks that could be useful in evaluating co‐production.**

The aim of this phase was to identify the most widely applied tools for evaluating co‐production. This process involved:

a. **Examining an initial set of papers** identified through the preparation of a protocol for this study [13], based on the knowledge of MK as an expert of participatory methods. These covered the theory and practice of public involvement and co‐production and this literature helped to identify and collate key terms related to co‐production, and provided further guidance on what tools and frameworks supporting the evaluation of co‐production could capture.
b. **Examining key models/frameworks/guidance:** building on (a), additional literature was sourced through non‐systematic searches for models and tools that evaluate approaches for involving people with lived experience. This approach was intended to be purposive to identify those most widely used, as opposed to comprehensive, and mainly involved ad hoc Google Scholar searches. We extracted and examined information on the scope and aims of these evaluation tools.
c. **Thematic interpretation of tools:** Once a small number of tools had been identified, a thematic approach was used to analyse the content and context of the tools. We followed parts of a process mirroring that laid out in [14]. This involved looking at each tool to explore whether there were similar characteristics found across each selected tool (patterns/repetition), and whether there were any discernible key terms/language/elements that would assist in developing a new evaluation tool, if the ones identified were not directly suitable.

This stage was not intended to be fully comprehensive or systematic but aimed to identify whether there were widely used tools for evaluating coproduction in research that could be used in our evidence synthesis research.

2. **Co‐producing the evaluation tool and process.**
  VB and MK delivered interactive workshops on evaluating co‐production, based on their learnings from earlier phases, as well as parallel work being undertaken by the Co‐production Collective [15]. VB is an expert in co‐production and MK is an expert in participatory methods and evidence synthesis. Co‐producers, who were part of a project described below, contributed their reflections on the findings and how to evaluate co‐production.
  Drawing on the initial exploration of selected existing tools, as well as engagement among the co‐production team, a first iteration of the tool was created in the format of a survey of open‐ended questions. Following the first iteration, a second iteration of the tool was created and discussed in a co‐production workshop, followed by a third iteration and further discussion. This third iteration was the evaluation tool that we would pilot to understand our own practice (see results).
3. **Piloting the tool and evaluation process in the project.**
  The tool was piloted in a project that sought to refine a theoretical framework that was to be used to guide evidence synthesis work [16]. Part of this piloting involved developing a process for how the tool would be administered, and this is outlined in the results.

## 3 Results

### 3.1 An Exploration of Co‐production Literature and Selected Frameworks/Tools

Sixteen papers were initially identified and examined for key terms related to PPI and co‐production (see supporting file for a list of studies and their features). These included a focus on co‐production (e.g., [4, 17, 18, 19, 20, 21, 22]) or public involvement more broadly (e.g., [22, 23, 24, 25, 26, 27, 28, 29, 30, 31, 32]). This literature gave us an understanding of core processes that could be considered when evaluating involvement and co‐production, with our focus being on evaluating against the values and principles of co‐production (see [4]). All the papers highlighted the importance of inclusive research designs and decision‐making. Several of the studies emphasised the importance of measuring the ‘impact’, ‘value’ or ‘effectiveness’ and the need to assess the tangible benefits of public and patient involvement, although there was less consensus on how to do this.

Next, five additional tools/frameworks were selected and were examined ([33, 34, 35, 36, 37]; see details in supplementary files). It was evident that the included tools represented guidance and resources oriented towards supporting the conduct and reporting of co‐production rather than evaluating process or change. Nevertheless, some of these tools and frameworks provided important signals on what could be considered when designing a tool to support evaluating co‐production. The themes identified from this analysis are summarised below in Table 1.

**Table 1:** Themes identified in the literature.

Process, values, or considerations to be captured in a reflective tool	Description
Positionality in knowledge sharing	If/how knowledge is being shared: is it through lived experience, a professional lens, or both, and how these are sensitively acknowledged and/or recorded.
Approach to co‐production	The stage, levels, and approaches that individuals are engaged in research highlights some delineation between co‐production and other forms of involvement and engagement. While co‐production could occur at any stage of research, co‐production may occur earlier than other forms of public involvement, and particularly around the scoping of the parameters of research.
Type of project and relationship with the project	Understanding the nature of the research topic is closely connected to how co‐production is approached. Different co‐production strategies may be adopted depending on factors such as sensitivity, available funding, and the research duration. As a result, the experience of co‐production can vary significantly between projects, such as emphasising active involvement in shaping the research and the co‐researcher role, compared with projects where the scope is more strictly predefined. Co‐producers may also wish to reflect on the value of their experiences and how these can contribute to broader outcomes.
Power relationship/sharing	Reflection on the degree of involvement and way in which decisions are made – how power in the decision‐making process is shared.
Feeling valued	Recording how a co‐producer perceives that their knowledge and expertise have been valued and integrated as someone who is part of the research team, rather than someone who provides their views on findings being reported back or part of an advisory group.

### 3.2 Development of Initial Tool

Based on the input from all co‐producers involved in workshops, it was deemed important to understand the rationale and expectation of all co‐producers' engagement and what motivates us as co‐producers to become involved; and then once immersed in the project, whether we feel listened to and valued. A summary of the areas captured in the tool are listed below (Table 2), and a copy of the full tool is available in the supporting materials.

**Table 2:** Areas of focus included in the tool (see supporting materials for a reproduction of the complete tool).

Area	Explanation
Positionality and expertise	Reflects how co‐producers share knowledge (personal, professional, other) and how their background influences their views (this was made topic specific).
Motivations and expected benefits	The motivations behind involvement (knowledge sharing, skills development, networking) and reasons for participation
Clarity of role and expectations	Assesses how clearly co‐producers understand their role and responsibilities, from the project's outset, along with suggestions for improvements.
Project involvement and contributions	Documents the practical aspects of involvement (research design, analysis, presentations, writing), including any additional roles co‐producers undertook.
Value and recognition	Explores co‐producers' perceptions of how valued their contributions were, considering acknowledgment and appreciation throughout the project.
Skills, knowledge, and personal growth	Examines how co‐production enhanced co‐producers' skills development.
Relationships and networking	Examines the strength and quality of connections formed and opportunities for meaningful collaboration
Comfort, support, and accessibility	Measures how comfortable, supported, and able co‐producers felt, noting changes and areas for improvement
Decision‐making and power sharing	Assesses perceptions of equity in power distribution and collaborative decision‐making processes, identifying effective practices and areas needing attention

### 3.3 Developing the Approach and Piloting the Tool

To measure the aspects of the co‐production process outlined in Table 2, we developed the following process for administering the tool:

1. **Generating data on individual perspectives:** We started by completing the evaluation tool and sharing these with an external evaluator (LR), who then created individual summaries of our responses. These summaries provided a personalised account of our experiences and perceptions.
2. **Individual Sense Check:** After the creation of summaries, each co‐producer was given the opportunity to review their individual report to clarify the content and overall understanding. We could highlight any omissions or request the removal of information we preferred not to disclose. An overarching document pooling all responses (anonymised as far as possible) was prepared and shared.
3. **Collective Sense Making:** A collective sense‐making session was held which involved collaborative reflection and dialogue. This session was critical as it moved us from our individual reflections to developing a collective understanding.

The tool was only trialled towards the end of the project, although the tool can be used to capture data much earlier in future projects to reflect evaluation and co‐production as a process that evolves throughout a project.

We reflected that the tool and process for administering the tool worked well, and we liked seeing our collective reflections from our individual reports as a prompt during collective sensemaking. We agreed that more continuous evaluation would allow us to implement changes (if any are needed) throughout the project. However, this may not always be the case and there may not be the same level of comfort in sharing experiences in future projects.

## 4 Discussion

In embedding co‐production as a central pillar of how we conduct evidence syntheses, our group were challenged by the lack of any obvious candidate tool and approach to help us understand the difference that co‐production makes. This is commensurate with the findings from other investigations [3, 4], and why others have also recently undertaken research on how to measure the ‘success’ of co‐production [15]. Our approach goes some way to addressing this.

In using the tool, most of the reflections we gathered were around the process of creating the theoretical framework (how we worked together). This is perhaps further testament of the value of the tool in measuring the *process* of co‐production, but there may be opportunity to further tease out the perceived value of co‐production to the *outcome* of research in future iterations.

We expect that both the tool and the approach will be co‐modified in future projects conducted by the LACES group and any other review teams drawing on this study. In particular, we expect future uses to be reflecting on the process of co‐production much earlier than was the case in our piloting.

## 5 Conclusion

The tool described here (which we refer to as the STRAPS tool – **S**ynthesising **T**hrough **R**eflection **A**nd **P**articipatory **S**ense‐making) provides a useful starting point for other review teams to evaluate co‐production in their reviews. It is imperative that we evaluate co‐production within evidence syntheses, and we encourage others to share their experiences of using the STRAPS tool with us.

## Author Contributions

**Meena Khatwa:** investigation; writing – review and editing; writing – original draft; methodology; formal analysis; conceptualization. **Vanessa Bennett:** investigation; methodology; writing – original draft; writing – review and editing; formal analysis; conceptualization. **Rachael C. Edwards:** investigation; writing – original draft; writing – review and editing; methodology; formal analysis; conceptualization. **Lisa Richardson:** formal analysis; methodology; writing – review and editing; writing – original draft; investigation; conceptualization. **Phuong Tu Nguyen:** investigation; methodology; writing – review and editing; writing – original draft; formal analysis. **Sajid Saleem:** investigation; writing – original draft; writing – review and editing; methodology; formal analysis. **Sylvia Chaires:** investigation; writing – review and editing; writing – original draft; formal analysis; methodology. **Alison O'Mara‐Eves:** investigation; writing – original draft; writing – review and editing; methodology; formal analysis; funding acquisition; conceptualization. **Dylan Kneale:** conceptualization; investigation; funding acquisition; writing – original draft; writing – review and editing; methodology; formal analysis.

## Conflicts of Interest

The authors declare no conflicts of interest.

## Supporting information

Supplementary file tool paper FIN REVISED CLEAN.

## Data Availability

The data that supports the findings of this study are available in the supporting material of this article.
