# Supplementary material for: A Co‐production Evaluation Tool Informed by Co‐production Workshops for Use in Evidence Synthesis Contexts
Source: Cochrane Evid Synth Methods. 2026 Jan 7;4(1):e70065. doi: 10.1002/cesm.70065 (PMC12782252; doi:10.1002/cesm.70065)
Supplement: Supplementary file 1 — Supplementary file tool paper FIN REVISED CLEAN. [file CESM-4-e70065-s001.docx]

Supplementary Materials

# Supplementary Materials 1 –

# STRAPS tool (Synthesising Through Reflection And Participatory Sense-making)

# STRAPS tool (Synthesising Through Reflection And Participatory Sense-making)

Evaluation reflection tool for co-production

This evaluation tool is intended to be completed by all co-producers.

**Please complete this form and return it to xxxx () by xxxxx.**

Expand all text boxes as needed.

## Expectations and experience

**In what capacity are you sharing your knowledge/expertise? (check all that apply)**

|  | Through living and learnt personal experience |
| --- | --- |
|  | Through living and learnt professional experience |
|  | Other (describe below) |

**Use this space to reflect on the personal, professional, and/or other knowledge, understanding, and experience you bring to this role (i.e., your positionality):**

**How has the experience you described above shaped your perceptions and beliefs about the topic of the research?**

## Benefits and motivations

**What did you want to gain from your contribution as co-producer? (check all that apply). Please also indicate which are your two main reasons for being involved (check up to 2).**

| **Check all that apply** | **Check up two 2 primary reasons** |  |
| --- | --- | --- |
|  |  | An opportunity to share my knowledge/experience/skills |
|  |  | Having an impact through the project outputs |
|  |  | Networking and making meaningful connections |
|  |  | Learning new skills/training |
|  |  | Identifying gaps for further project development |
|  |  | Working collaboratively |
|  |  | An opportunity to create better research |
|  |  | An opportunity to produce research in a more equitable way |

**Use the space below to describe anything else (in addition to the above) you hoped to gain from your involvement in this project and what motivated you to become involved:**

## Co-production evaluation

**Type of involvement**

**What did your role on this project involve? (check all that apply)**

|  | Design of research |
| --- | --- |
|  | Preparing information |
|  | Sharing knowledge ideas |
|  | Presenting |
|  | Helping with analysis |
|  | Writing up findings |
|  | Other (please describe below) |

**In what other ways were you involved in the project?**

## Clarity of role and expectations

**How clear were you on what your role was when the project began?**

| Not at all clear | | | | | Extremely clear | | | | |
| --- | --- | --- | --- | --- | --- | --- | --- | --- | --- |
| 1 |  |  |  |  |  |  |  |  | 10 |

**How clear were you on what was expected of you when the project began?**

| Not at all clear | | | | | Extremely clear | | | | |
| --- | --- | --- | --- | --- | --- | --- | --- | --- | --- |
| 1 |  |  |  |  |  |  |  |  | 10 |

**Use this space to reflect on the clarity of the role, and what is expected of you. Is there anything that could be clearer and how do you think this could have been communicated? Is there anything about the role that has been communicated particularly well?**

## Expectations

**How well has the project met your expectations so far?**

| Not at all | | | | | Extremely well | | | | |
| --- | --- | --- | --- | --- | --- | --- | --- | --- | --- |
| 1 |  |  |  |  |  |  |  |  | 10 |

**How well has the role met your expectations for the project since you started?**

| Not at all well | | | | | Extremely well | | | | |
| --- | --- | --- | --- | --- | --- | --- | --- | --- | --- |
| 1 |  |  |  |  |  |  |  |  | 10 |

**Please describe the ways in which the project and role met and/or failed to meet your expectations. Did the extent to which it met your expectations improve or decline over time? What was done well and what could be improved?**

## Value

**How well do you feel that your input was valued?**

| Not at all | | | | | Extremely well | | | | |
| --- | --- | --- | --- | --- | --- | --- | --- | --- | --- |
| 1 |  |  |  |  |  |  |  |  | 10 |

**Please describe the ways in which your input was valued and/or not valued? Did the extent to which your input was been valued improve or decline over time? What was done well and what could be improved?**

## Motivation

**How motivated did you feel about your involvement with the project?**

| Not at all motivated | | | | | Extremely motivated | | | | |
| --- | --- | --- | --- | --- | --- | --- | --- | --- | --- |
| 1 |  |  |  |  |  |  |  |  | 10 |

**Please describe how motivated you have felt over the course of the project? Has this changed over time? What has been done well and what could be improved to help you feel more motivated?**

## Skills and knowledge

**To what extent were your skills and knowledge enhanced through your involvement with this project?**

| Not at all enhanced | | | | | Greatly enhanced | | | | |
| --- | --- | --- | --- | --- | --- | --- | --- | --- | --- |
| 1 |  |  |  |  |  |  |  |  | 10 |

**Please describe any new skills or knowledge you have gained. Did this change over time? Are there any skills/knowledge that you wanted to learn but didn’t have the opportunity?**

## Connections

**To what extent did you make meaningful connections through your involvement in this project?**

| No connections made | | | | | Strong connections made | | | | |
| --- | --- | --- | --- | --- | --- | --- | --- | --- | --- |
| 1 |  |  |  |  |  |  |  |  | 10 |

**Please describe the strength of any connections you have made. Did this change over time? What was done well and what could have be improved to help you make more meaningful connections?**

## Comfort and environment (accessibility)

**How comfortable did you feel contributing to the project?**

| Not at all comfortable | | | | | Extremely comfortable | | | | |
| --- | --- | --- | --- | --- | --- | --- | --- | --- | --- |
| 1 |  |  |  |  |  |  |  |  | 10 |

**Please describe why you felt comfortable or not making contributions to the project. Did this change over time? What was done well and what could have been improved to help you feel more comfortable? Were any needs you may have in terms of accessibility met?**

## Decision making and power sharing

**Did you feel that power was shared equally over the duration/length of the project?**

| Not at all equal | | | | | Extremely equally | | | | |
| --- | --- | --- | --- | --- | --- | --- | --- | --- | --- |
| 1 |  |  |  |  |  |  |  |  | 10 |

**Please describe why you feel that power was shared more or less equally? Did this change over time? What was done well and what could have been improved to enhance equality in power sharing?**

## Support

**How supported did you feel in your role?**

| Not at all supported | | | | | Extremely supported | | | | |
| --- | --- | --- | --- | --- | --- | --- | --- | --- | --- |
| 1 |  |  |  |  |  |  |  |  | 10 |

**Please describe why you felt more or less supported? Did this change over time? What was done well and what could have been improved to help you feel more supported?**

## Any other comments

**Please use the box below to add in any reflections you’d like to make on being a co-producer on this project.**

**Reflections on the evaluation tool**

**Please use the box below to add any reflections on this evaluation tool. For example: How easy was it to complete? Were there any questions missing?**

# Supplementary Materials 2 – List of studies included in the review and the values that could be included in an evaluation

Table 2: Overview of studies included key search terms

| Author | Title of paper | Focus of paper/report | Keywords/codes and or values |
| --- | --- | --- | --- |
| (N=16) | | | |
| Allen, Cree et al. (2020)^[[1]](#footnote-1)^ | Exploring patient and public involvement (PPI) and co-production approaches in mental health research: learning from the PARTNERS2 research programme | paper explores the successes and challenges encountered by one research team on PPI in research | Experiential expertise  Collaborative methods  Service users’ researchers  Cooperative inquiry  Emotional work  Safe space  Understanding challenges  Personal identities  **Power sharing**  Assessing progress |
| Boote, Baird and Beecroft (2010) | Public involvement at the design stage of primary health research: a narrative review of case examples. | To review published examples of public involvement in research design, to synthesise the contributions made by members of the public, as well as the identified barriers, tensions and facilitating strategies. | Consent procedures  Outcome suggestions  Consultation  Collaboration  ‘User control’^[[2]](#footnote-2)^  Experiential insights  Involvement in research design  Advice and timing  Barriers and tensions  Time implications  Cost implications  Representativeness  Language and jargon  Facilitating strategies  Cultural sensitivity |
| Dawson, Ruddock et al. (2020) | Patient and public involvement in doctoral research: reflections and experiences of the PPI contributors and researcher | Detailed account of how PPI was embedded in a doctoral research project, PPI contributors and researcher’s reflection key recommendations in involving people | Prioritising research topic  Reflections  Impact  Expectations  Reviewing and commenting  Co-authoring  Designing  Dissemination  Implementation  Plain English  Respect  Support  Transparency  Responsiveness  Fairness  Opportunity  **Accountability** |
| Durose, Beebeejaun et al. (2012) | Towards Co-production in research with communities | This review takes a cross-cutting perspective, aiming to advance the theory and practice of coproduction in research with communities. | Research  Community  Coproducers  Power  Presence  Marginalisation  Authenticity  Beyond text  Interactive knowledge  **Accountability**  Public value  Reflexivity |
| Florin and Dixon (2004) | Public involvement in health care | The government is promoting public involvement in health care as part of new NHS policy.1,2 Proposals include allowing the public to elect members of the governing boards of foundation trusts3 and primary care trusts' obligations to engage with the public.4  However, clarity and consensus are lacking about what public involvement means in health care, why it is desirable, and whether current policies will meet the desired objectives. We examine the latest polices and their potential effects. | Shape services  Democratic decision making  **Accountability**  Clear aims  Clarity and consensus |
| Gerlak, Guido et al. (2023) | Stakeholder engagement in the co-production of knowledge for environmental decision-making | A systematic review focusing on collaborative engagement between scientist and decision makers. The authors explore how stakeholders are defined, the processes by which stakeholders are engaged, the societal impacts associated with stakeholder engagement, and the barriers and enablers to stakeholder engagement. | Stakeholders  Communities  Impact  Environment  Power  Communication  Project framing  Activities :  Workshops  Conferences  Distributing information  Art based  Contractual  Collaborative  Consultative  Collegial^[[3]](#footnote-3)^  Indigenous ^[[4]](#footnote-4)^ |
| Greenhalgh, Hinton et al. (2019) | Frameworks for supporting patient and public involvement in research: systematic review and co-design pilot | To identify and synthesis published framework consider whether and how these have been used and apply design principles and usability | Co-design  Frameworks  Power focused  Challenging power  Power sharing  Priority setting research  Transparency  Diversity  Study focused  Report focused  Audit trail  Partnership focused  training |
| McCoy, Warsh et al. (2019) | Patient and public involvement: Two sides of the same coin or different coins altogether? | Clarifying meaning and justification for PPI split apart drawing distinction between them | Patient involvement  Public engagement  Public involvement  First-hand experience  Consumers  Service users  Survivors  Members of relevant population  Impartiality  Citizens’ jury |
| O'Mara-Eves, Laidlaw et al. (2022) | The Value of Co-production Research project: A rapid critical review of the evidence | What are the different types of co-production within research and what are their value(s)? | Values  Co-authored  Rich co-production  Complex intervention  Transparent  Inclusive  Equality  Diversity  Accessibility  Reciprocity |
| Phillips, Christensen-Strynø and Frølunde (2022)^[[5]](#footnote-5)^ | Arts based co-production in participatory research: harnessing creativity in the tension between process and product | The aim is to present and illustrate the use of a theoretical framework for analysing the complexities of co-production in the nexus between arts and research – with a focus on the overarching tension between cultivating the collaborative, creative process and producing specific research results. | Arts based  Co-production  Affective  Aesthetic knowing  Participatory research  Relational practices  Mutual learning  Mutual caring  **New knowledge**  **Destabilizing of knowledge** hierarchy  Transformative practice |
| Reed, Stringer et al. (2014) | Five principles for the practice of knowledge exchange in environmental management | This paper outlines five principles for effective practice of knowledge exchange, which when applied, have the potential to significantly enhance the impact of environmental management research, policy and practice. The paper is based on an empirical analysis of interviews with 32 researchers and stakeholders across 13 environmental management research projects, each of which included elements of knowledge co-creation and sharing in their design. | Design  Co-design  Engage  Represent  Impact  Reflect & sustain  Effective knowledge  Diversity  Trust  Flexibility  **Knowledge exchange**  **Knowledge brokers**  Good practice |
| Snape, Kirkham et al. (2014) | Exploring perceived barriers, drivers, impacts and the need for evaluation of public involvement in health and social care research: a modified Delphi study | To explore areas of consensus and conflict in relation to perceived public involvement (PI) barriers and drivers, perceived impacts of PI and ways of evaluating PI approaches in health and social care research. | Tokenism  PI valuable contribution  Building partnerships  Barriers and drivers  Impact and value of PI  Intrinsic value  Clear guidance for challenges  Models of good practice  Measurable standards |
| Staley (2015) | Is it worth doing? Measuring the impact of patient and public involvement in research | This review reflects on the use of quantitative approaches to evaluating  impact. It concludes that the statistical evidence is weakened by not paying  sufficient attention to the context in which involvement takes place and the way it is  carried out. | Service user involvement  Consumer involvement  Measuring impact  Evidence  **Experiential knowledge**  Planning  Values  Impacts  Research ethics  Implementation for change |
| Staniszewska, Brett et al. (2017) | GRIPP2 reporting checklist: tools to improve reporting of patient and public involvement in research | To develop international consensus on the key items to report to enhance the quality, transparency, and consistency of the PPI evidence base. To collaboratively involve patients as research partners at all stages in the development of GRIPP2. | Long and short form versions^[[6]](#footnote-6)^  Stages and nature of involvement  Context  Measurement of impact  Outcomes  Economic assessment  Reflections |
| Turnhout, Metze et al. (2020) | The politics of co-production: participation, power, and transformation. | The literature review focuses on political and power dimensions of co-production (or not) and how these dimensions have affected the outcomes of co-production | Empowerment  **power relations**  Global South & North  Colonialism  Western bias  Trust  Depoliticization  None-elite participants  Horizontal relationships  **Actionable knowledge** |
| Williamson (2014) | A Response to the Open Peer Commentaries on  “Patient and Citizen Participation in Health: The Need for Improved Ethical Support” | Appropriate ethical support patient and citizenship involvement  Making clear distinction between patient and public (citizen) | Involvement  Autonomy  Relational autonomy  Experience  Viewpoints  Relational exchange  democratic |

## Supplementary Materials 3 – Overview of selected tools for evaluating co-production

Table 3: Overview of selected tools for evaluating co-production

| Tool | Features | Implications for evaluating co-production |
| --- | --- | --- |
| NHS England’s ‘A Co-production Model’ (2016) | States that five values are needed to ensure that co-production is enacted ((1) ownership, understanding and support of co-production by all; (2) commitment to sharing power and decisions; (3) a culture in which people are valued and represented; (4) a culture of openness and honesty; (5) clear communication in plain English.  Outlines seven recommended steps for facilitating co-production including (i) getting agreement from senior leaders to champion co-production; (ii) use open and fair processes to recruit co-producers; (iii) put systems in place to recognise contributions; (iv) involve citizens from the outset; (v) build co-production into activities until it becomes the status quo; (vi) train staff and citizens in co-production; (vii) regularly review progress. | Could be adapted into a checklist to assess if co-production happened. Less clarity on how the seventh step can be enacted and the tools available to support reviewing progress and evaluating the implementation and impact of co-production. |
| NIHR (Denegri, Coldham et al. 2015) | NIHR Involve guidance sets out ways of involving the public throughout the research cycle, including in evaluation. In the context of co-production, through adapting the work of Boyle, Slay and Stephens (2010), the framework sets out a series of principles for putting co-production into practice including: (1) Building on people’s existing capabilities; (2) Promoting mutuality and reciprocity; (3) Developing peer support networks; (4) Breaking down boundaries; (5) Facilitating as well as delivering; (6) Recognising people and their experiences as assets. | This guidance supports public involvement including, but not specific to, co-production. Principles could be adapted into a checklist to assess if and how well co-production happened. |
| Cancer Research UK’s Patient Involvement Toolkit for researchers (CRUK 2023) | Recognise a spectrum of approaches from *Participation* (which can include patients or healthy volunteers take part in research); to Engagement (where knowledge is shared with the public); through to *Involvement* (whereby patients’ experiences can help shape research, with an emphasis placed on research being conducted with or by the patients rather than to them). For involvement to be meaningful, CRUK recommend adopting NIHR Involve principles including: Respect; Support; Transparency; Responsiveness; Fairness of opportunity; and Accountability. | This guidance supports public involvement including, but not specific to, co-production. Principles could be adapted into a checklist to assess if and how well co-production happened. |
| Parkinsons UK PPI guidance of for researchers (2024) | This guidance mirrors the language of the CRUK approach above, and recognises that co-production is one method of involving patients. They place emphasis on ‘meaningful involvement’ (through planning and understanding)’ and ‘adding value’ to research. The approach taken by Parkinson’s UK is to bridge the gap between researchers to help ‘connect’ with people affected by Parkinson’s and develop working relationships with PPI volunteers. They illustrate that people affected by Parkinson’s can be involved throughout all stages of the research cycle from the initial stages of identifying and prioritising the focus of the research through to evaluating the research. Within their guidance, they stress the importance of ‘sharing of power and responsibility’ through ongoing dialogue and joint ownership of research. | This guidance supports public involvement including, but not specific to, co-production. Principles could be adapted into a checklist to assess if and how well co-production happened. |
| Homeless Link: Co-Production toolkit (2022) | Homeless Link underscore the importance of sharing power in their toolkit, viewing co-production as an asset based approach that builds on the skills of all individuals through developing equal relationships. They set out a number of principles of co-production including: (1) include everyone; (2) listen; (3) communicate clearly; (4) encourage development; (5) take action; (6) look at the big picture; (7) open all doors; and (8) trust and respect. | This guidance is specific to co-production. Principles could be adapted into a checklist to assess if and how well co-production happened. |

# References

Allen, D., L. Cree, P. Dawson, S. El Naggar, B. Gibbons, J. Gibson, L. Gill, R. Gwernan-Jones, C. Hobson-Merrett and B. Jones (2020). "Exploring patient and public involvement (PPI) and co-production approaches in mental health research: learning from the PARTNERS2 research programme." Research Involvement & Engagement **6**(1).

Boote, J., W. Baird and C. Beecroft (2010). "Public involvement at the design stage of primary health research: a narrative review of case examples." Health policy **95**(1): 10-23.

Boyle, D., J. Slay and L. Stephens (2010). Public services Inside Out: Putting co-production into practice. London, NESTA.

CRUK. (2023). "Patient involvement toolkit for researchers." Retrieved 16/2/25, 2025, from <https://www.cancerresearchuk.org/patient-involvement-toolkit-for-researchers>.

Dawson, S., A. Ruddock, V. Parmar, R. Morris, S. Cheraghi-Sohi, S. Giles and S. Campbell (2020). "Patient and public involvement in doctoral research: reflections and experiences of the PPI contributors and researcher." Research involvement and engagement **6**: 1-13.

Denegri, S., T. Coldham, S. Eglin, R. Frost, L. Kerridge, R. Matthews, S. Staniszewska, C. Stephenson, D. Stewart, P. Yeeles, S. Buckland, K. Pattison and K. Mann (2015). Going the extra mile: improving the nation’s health and wellbeing through public involvement in research. London, NIHR.

Durose, C., Y. Beebeejaun, J. Rees, J. Richardson and L. Richardson (2012). "Towards co-production in research with communities."

Florin, D. and J. Dixon (2004). "Public involvement in health care." Bmj **328**(7432): 159-161.

Gerlak, A. K., Z. Guido, G. Owen, M. S. R. McGoffin, E. Louder, J. Davies, K. J. Smith, A. Zimmer, A. M. Murveit and A. Meadow (2023). "Stakeholder engagement in the co-production of knowledge for environmental decision-making." World Development **170**: 106336.

Greenhalgh, T., L. Hinton, T. Finlay, A. Macfarlane, N. Fahy, B. Clyde and A. Chant (2019). "Frameworks for supporting patient and public involvement in research: systematic review and co‐design pilot." Health expectations **22**(4): 785-801.

Homeless Link. (2022). "Co-production Toolkit " Retrieved 16/2/25, 2025, from <https://homeless.org.uk/knowledge-hub/co-production-toolkit/>.

McCoy, M. S., J. Warsh, L. Rand, M. Parker and M. Sheehan (2019). "Patient and public involvement: Two sides of the same coin or different coins altogether?" Bioethics **33**(6): 708-715.

NHS England and Coalition for Collaborative Care (2016). A Co‐production Model Five Values and Seven Steps to Make This Happen in Reality. London, NHS England.

O'Mara-Eves, A., L. Laidlaw, B. Candy, C. Vigurs, A. Collis and D. Kneale (2022). The Value of Co-Production Research Project: A Rapid Critical Review of the Evidence. London, Co-production Collective, UCL.

Parkinson's UK. (2024). "Patient and Public Involvement in research." Retrieved February 16th, 2025, from <https://www.parkinsons.org.uk/research/patient-and-public-involvement-research>.

Phillips, L., M. B. Christensen-Strynø and L. Frølunde (2022). "Arts-based co-production in participatory research: harnessing creativity in the tension between process and product." Evidence & Policy **18**(2): 391-411.

Reed, M. S., L. C. Stringer, I. Fazey, A. C. Evely and J. H. Kruijsen (2014). "Five principles for the practice of knowledge exchange in environmental management." Journal of environmental management **146**: 337-345.

Snape, D., J. Kirkham, N. Britten, K. Froggatt, F. Gradinger, F. Lobban, J. Popay, K. Wyatt and A. Jacoby (2014). "Exploring perceived barriers, drivers, impacts and the need for evaluation of public involvement in health and social care research: a modified Delphi study." BMJ open **4**(6): e004943.

Staley, K. (2015). "‘Is it worth doing?’Measuring the impact of patient and public involvement in research." Research involvement and engagement **1**: 1-10.

Staniszewska, S., J. Brett, I. Simera, K. Seers, C. Mockford, S. Goodlad, D. Altman, D. Moher, R. Barber and S. Denegri (2017). "GRIPP2 reporting checklists: tools to improve reporting of patient and public involvement in research." bmj **358**.

Turnhout, E., T. Metze, C. Wyborn, N. Klenk and E. Louder (2020). "The politics of co-production: participation, power, and transformation." Current opinion in environmental sustainability **42**: 15-21.

Williamson, L. (2014). "Patient and citizen participation in health: the need for improved ethical support." The American journal of bioethics **14**(6): 4-16.

1. Many authors part of PARTNERS2 research programme [↑](#footnote-ref-1)
2. where the public designs and undertakes the research and where researchers are only invited to participate at the invitation of the public [↑](#footnote-ref-2)
3. Collegial: Stakeholders and researchers work together; decisions are made by stakeholders or by consensus of the team. [↑](#footnote-ref-3)
4. Indigenous :Research process is centered in indigenous value systems and historical contexts; stakeholders hold decision-making authority. [↑](#footnote-ref-4)
5. See page 395 of article for illustrative table ‘contested discursive terrain of arts-based co-production’ [↑](#footnote-ref-5)
6. Long and short form aim to improve quality, transparency and consistency of international PPI evidence base [↑](#footnote-ref-6)
